# Case Report: Rare Case of Synchronous Neck Metastasis From Metachronous Bilateral Renal Cell Carcinoma

**DOI:** 10.3389/fonc.2021.677714

**Published:** 2021-07-27

**Authors:** Jun Dai, Chen Fang, Xiaoqun Yang, Xin Huang, Wei He, Chenghe Wang, Juping Zhao, Fukang Sun

**Affiliations:** ^1^Department of Urology, School of Medicine, Ruijin Hospital, Shanghai Jiao Tong University, Shanghai, China; ^2^Department of Pathology, School of Medicine, Ruijin Hospital, Shanghai Jiao Tong University, Shanghai, China

**Keywords:** bilateral, MTSCC, PRCC, gene detection, neck metastasis, case report

## Abstract

Renal cell carcinoma (RCC) is a malignant tumor that can metastasize easily. Hence, many patients have already developed metastasis when they are diagnosed. It is also one of the most common tumors that metastasize to the head and neck through extranodal disease. Herein, we reported a case of a 53-year-old man with cervical metastasis from bilateral RCC. Interestingly, whole exome sequencing (WES) and clonal evolution analysis revealed that bilateral renal tumor lesions and neck metastases (squamous cell carcinoma) share the same subclones and a large number of gene variants, while the pathological morphology is different (left nephrotic foci, a mixed pattern of mucinous tubular and spindle cell carcinoma (MTSCC) with papillary adenoma; right renal foci, papillary renal cell carcinoma (PRCC)). This was first reported in RCCs to the best of our knowledge. This case suggests that genotype analysis can be a powerful supplementary examination for clinical histopathological diagnosis. Gene detection has great significance for the accurate diagnosis and treatment of RCC metastasis or multiple lesions.

## Introduction

Renal cell carcinoma (RCC) is the third most common metastatic tumor that metastasizes to the head and neck, after lung cancer and breast cancer (1). Metastasis of RCC to the head and neck area has been demonstrated primarily in the paranasal sinuses, parotid gland, mandible, larynx and hypopharynx. RCC should be suspected whenever a metastatic lesion is encountered in the head and neck area, even if the metastatic lesion is the first clinical presentation (2). The diagnosis of metastatic RCC should be suspected in any patient even with a remote history of RCC. Several cases of head and neck metastasis of RCC have been reported in the literature, but the genetic characteristics and cloning analysis of the primary and metastatic lesions have not been reported. The case reported herein was characterized by bilateral heterochronous renal tumors, in which the third occurrent lesion first appeared in the right neck.

## Case Presentation

### Clinical Findings

**Figure 1:**
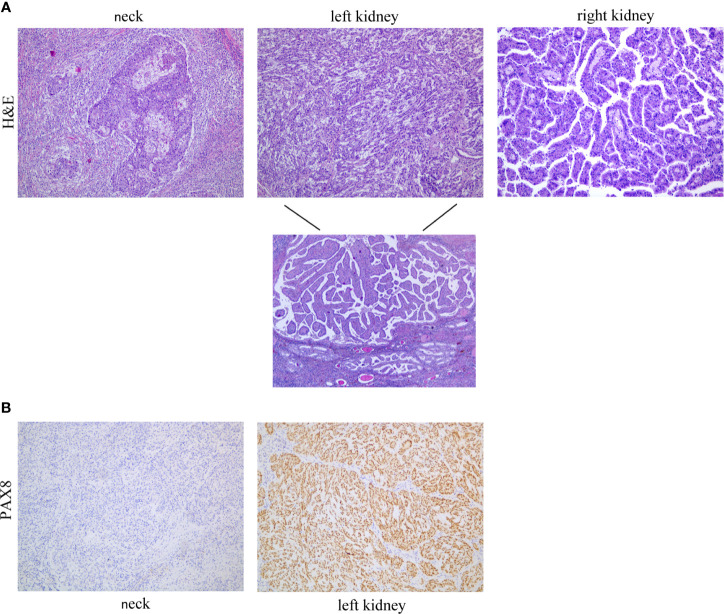
**(A)** Pathological examination of cervical mass revealed squamous cell carcinoma (left panel); pathological findings for the renal tumor were a mixed pattern of mucinous tubular and spindle cell carcinoma (MTSCC) with papillary adenoma in the left kidney (middle panel); and a papillary renal cell carcinoma (PRCC) in the right kidney (right panel). **(B)** Neoplastic cells in the neck and left kidney showed negative and positive staining for PAX8, respectively.

In June 2019, a 53-year-old man was admitted to a local hospital due to a palpable “right neck mass”. He had no fever, dizziness, fatigue, abdominal pain, backache, frequent urination, urgency, or changes in urine color. There was no other positive clinical sign on physical examination. No family history of hereditary diseases was detected. After surgical resection, a metastatic squamous cell carcinoma was diagnosed pathologically (**Figure 1A**, left panel). In the following PET-CT (Positron Emission Tomography-Computed Tomography) scan showed nodular iso-density foci in the upper pole of the left kidney, with a diameter of about 28.5 mm, SUVmax value of 8.3, increased FDG (fluorodeoxyglucose) metabolism, indicative of malignant lesions, and semi-oval infarct foci in the center of the right kidney. On July 29, 2019, renal puncture was performed, and the pathological diagnosis was epithelial tumor based on the immunohistochemical positive results of relevant markers. SPECT/CT (Single Photon Emission Computed Tomography) showed no obvious abnormalities in blood flow perfusion, uptake and excretion in both kidneys.

**Figure 2:**
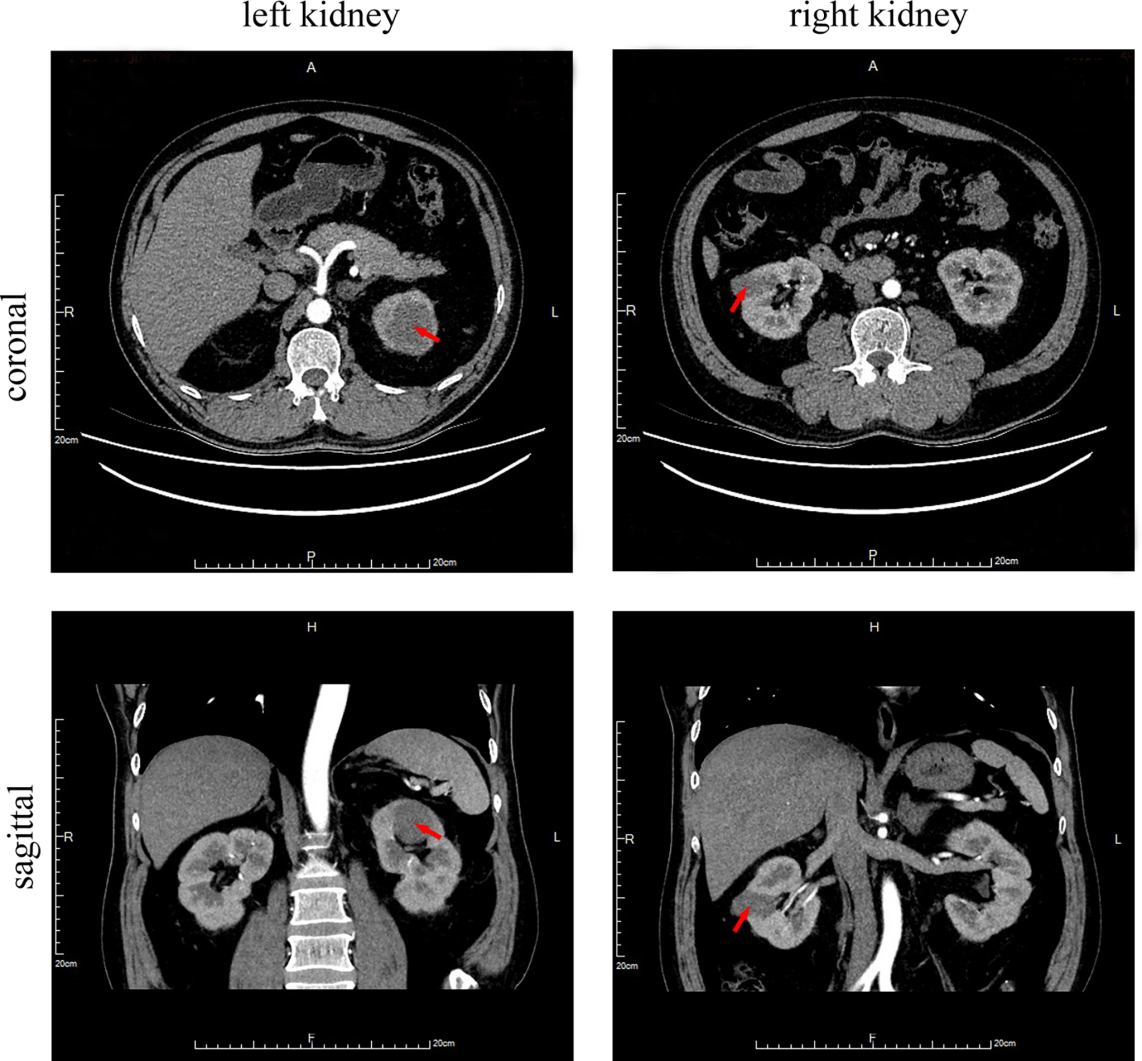
Abdomen CT scan with contrast media. Circular iso-density lesions of about 4.4 * 4.2 cm in the upper left kidney (arrow) and an oblong iso-density foci of about 3.4 * 2.1 cm in the middle of the right kidney (arrow).

For further surgical treatment, the patient was transferred to our hospital on August 12, 2019. The renal artery CTA (Computed Tomographic Angiography) results showed an oblong iso-density foci of about 3.4 * 2.1 cm in the middle of the right kidney, and a 4.4 * 4.2 cm-like circular iso-density lesion in the upper left kidney (**Figure 2**). The local boundary was unclear from the adjacent renal parenchyma. It was slightly protruded on the surface of the renal parenchyma, and the lesion was slightly uneven and enhanced by enhanced scan. There was no obvious abnormality in the diagnosis of bilateral renal artery CTA. Kidney magnetic resonance imaging (MRI) confirmed a 2.9 * 1.5 cm circular abnormal signal lesion in the middle of the right kidney and a 3.6 cm diameter circular abnormal signal lesion in the upper pole of the left kidney, which was isointense on T1-weighted imaging (T1WI), a low signal area on fST2WI, and hyperintense on DWI. The signal of the ADC map was reduced, and a slight uneven enhancement was seen by enhanced scan. Based on the above findings, the possible diagnosis was a malignant tumor of the kidney.

On August 19, 2019, a laparoscopic radical nephrectomy (left) was performed under general anesthesia. On December 12, 2019, the patient was admitted to the hospital due to a “right kidney mass”. After completing the relevant examinations, the “Da Vinci Robot-Assisted Laparoscopic Partial Right Nephrectomy” was successfully performed. No complication was observed and the patient was discharged six days after surgery. The patient’s condition currently remains stable. PET-CT scan re-examination found no local recurrence or distant metastasis one year after the operation.

### Pathological and Genetic Findings

Histological examination of the radical nephrectomy specimen of the left kidney showed a mixed pattern of mucinous tubular and spindle cell carcinoma (MTSCC) with papillary adenoma (**Figure 1A**, middle panel). Microscopy examination of the partial nephrectomy of the right kidney revealed a papillary renal cell carcinoma (PRCC) (**Figure 1A**, right panel). The histological morphology and immunohistochemical results of renal tumor were different from those of neck tumor. PAX8 staining showed positive expression (**Figure 1B**).

**Figure 3:**
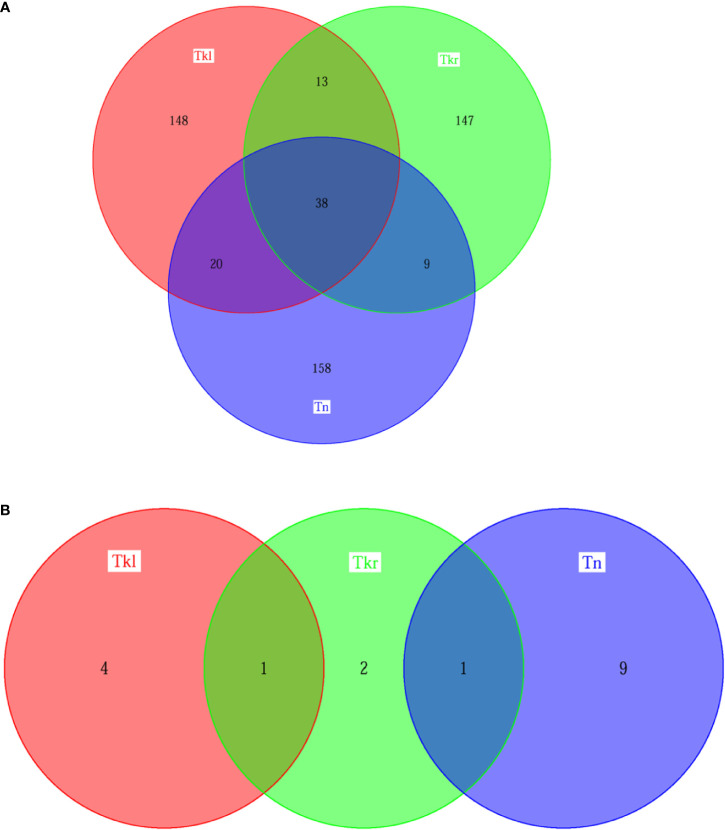
Similarity among different lesions based on somatic mutation analysis. **(A)** Venn diagram illustrating the distributions of genetic mutation in different lesions [left kidney (Tkl) *vs* right kidney (Tkr) *vs* neck (Tn)]. **(B)** Venn diagram illustrating the distributions of driver genetic mutation in different lesions [left kidney (Tkl) *vs* right kidney (Tkr) *vs* neck (Tn)].

To further investigate their genetic characteristics, whole exome sequencing (WES) was performed separately. A total of 304 putative somatic variants of 225 genes were identified in neck tumor tissues, of which 10 were identified as driver gene mutations, including CDKN2A, KMT2D and NSD1 mutations that are common drivers of head and neck squamous cell carcinoma (HNSC) (3). Similarly, the WES results of renal tumor samples revealed 361 somatic variants in 219 genes in the left kidney and 270 somatic variants in 207 genes in right kidney, including five and four driver gene variants, respectively (**Figure 3** and **Supplementary Figure 1**). The three lesions have both private and shared mutation genes shown in **Figure 3**. Similarly, 51, 58 and 47 shared gene variants were detected respectively between left & right kidney, left kidney& neck, and right kidney & neck. Thirty-eight common mutated genes were shared in all three tumor tissues. For the driver gene mutations, the right nephropathy foci shared one driver gene mutation with the left kidney (WT1) and the neck lesions (CREBBP), respectively. But no driver mutated gene was shared in all three tissues (**Figure 3B**).

**Figure 4:**
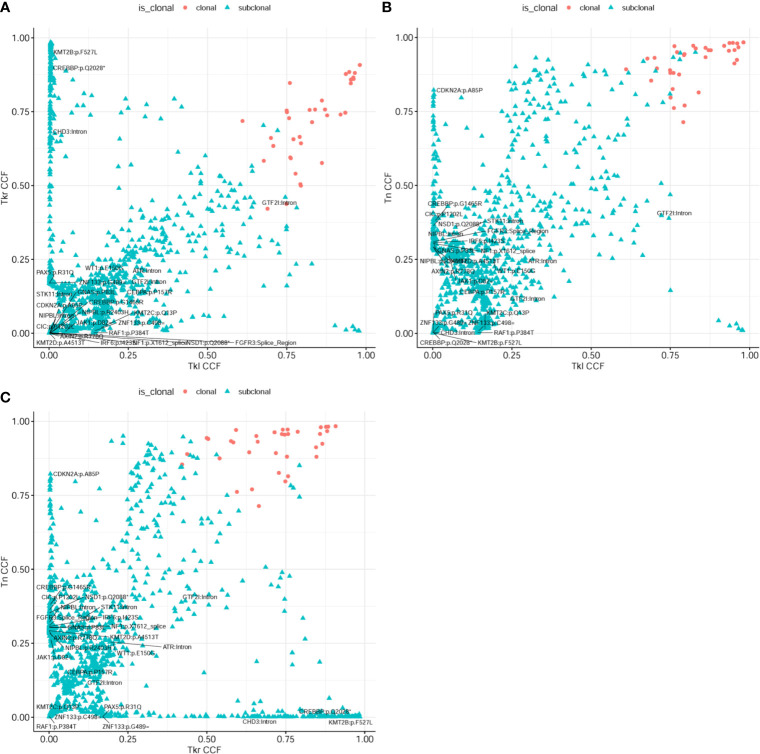
Analysis of heterogeneity between different lesions. **(A)** Two-dimensional analysis of tumor subclonal architecture in right kidney (Tkr) and left kidney (Tkl); and **(B)** in neck (Tn) *vs*. left kidney (Tkl); and **(C)** in neck (Tn) *vs*. right kidney (Tkr). These subclones were shared between the spatially distinct lesions.

**Figure 5:**
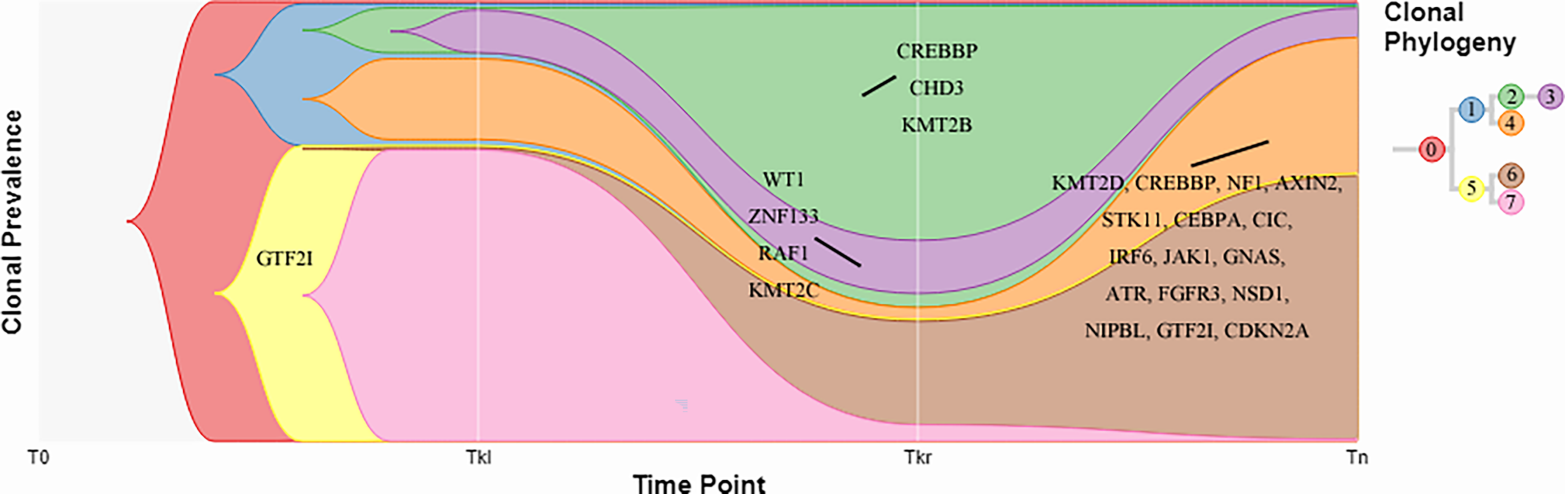
Fish plots constructed by timescape. Colors indicate different clones. Driver genes detected in the clones are shown. Tkl, left kidney tumor; Tkr, right kidney tumor; Tn, neck tumor.

In addition, a clonal evolution analysis was performed to examine the heterogeneity of tumor evolution among different lesions. We used a previously reported hierarchical Bayesian clustering model (4) to define clones and subclones based on clusters of SNVs sharing similar cancer cell fractions (CCF). CCF distributions are shown in **Figure 4**. These clones and subclones were shared between the spatially distinct lesions. Correspondent clonal components also existed in different lesions, especially some driver gene mutations, such as NFKB2, WT1, GTF2I, etc. The clone evolution analysis showed that the clone components of the initial left renal primary lesion and the final cervical squamous cell carcinoma lesion were different. The left renal tumor was mainly composed of clones 3, 4, and 7, the right renal tumor was mainly composed of clones 2, 3, and 6, while the cervical mass was mainly composed of clones 4 and 6 (**Figure 5**). Therefore, the primary clones in the metastatic lesions may not be derived from the primary clones in the left kidney tumor. Instead, they may have originated from the subclones generated before the diagnosis. Some driver gene variants detected in clones with different lesions were unique and some were shared. The putative dynamic mutation profiles during the disease course are represented in **Figure 5**. These results can serve as a strong complement to histopathological findings, suggesting that these lesions may have evolved from the original focus, although there are differences in the pathological morphology of different lesions.

## Discussion

Renal cell carcinoma (RCC) is a malignant tumor that can easily metastasize, and many patients already have metastasis when they are diagnosed. RCC is thought to be the third most common subclavian tumor to metastasize to the head and neck. Supraclavicular metastases were found in 3% (22/671) of RCC patients. The transfer rate has been reported to be as high as 15% (5–7). Our patient initially presented with symptomatic metastasis of a “right neck mass” in the absence of an evident primary tumor. Subsequent PET-CT showed lesions in the kidney and suggested the possibility of malignant tumors. Microscopy observation of postoperative specimens showed that the neck tumor was metastatic squamous cell carcinoma, while the microscopic pathology of the left kidney lesion showed the presence of mixed cells composed of spindle cells, tubules and papillary parts, but no squamous cells, although we could not rule out whether this was the interference of intratumoral heterogeneity. After a few months, the patient underwent a laparoscopic partial nephrectomy for resection of a mass in the right kidney. The pathology revealed PRCC. We hypothesized that there might be a possibility of metastasis between the bilateral renal neoplasms due to their similar pathological components (both having papillary parts), but for neck tumor, we cannot determine the evolutionary relationship pathologically. A case of bilateral RCC with similar pathology was reported in 2017 (8). The patient developed multiple bone metastases of MTSCC two years after bilateral nephrectomy for PRCC, in which the tumor in the right kidney was diagnosed as mucus-deficient MTSCC and the tumor in the left kidney was diagnosed as PRCC. Another report described a case with RCC nasal metastases in a metachronous bilateral neoplasm. Ten years after left nephrectomy, the second concealed lesion first appeared in the ipsilateral nasal metastasis, and the tumor lesions were also subsequently found in the right kidney (1). Unfortunately, there was no categorical comparison of pathological morphology between lesions in the above case report. In our paper, WES detection and clonal evolution analysis were performed subsequently to further explore the evolutionary heterogeneity of tumor, given the difficulty in identifying the intrinsic links between different lesions in pathological morphology, especially those in the neck.

Few reports have simultaneously explored the intra-tumor heterogeneity in histomorphology and molecular subtype between primary and paired metastatic tumors, especially in patients with head and neck metastases from bilateral renal carcinoma. To the best of our knowledge, this is the first report on spatially different lesions that share the same subclones, indicating that these lesions may originate from a common ancestor, although different lesions have differences in pathological morphology. Similar reports have been published in other cancers. Zhu et al. (9) and Zhao et al. (10) reported cases with different primary sites and metastatic tissue morphologies, but the same clonality in ALK-rearranged lung adenocarcinoma. Moreover, in a case of bronchial melanoma metastasis, Karpathiou et al. (11) suggested that the metastasis is always different compared to the primary tumor, so a negative result (different from the primary tumor) does not exclude metastasis. In our study, there were 38 mutated genes shared in all three tumor tissues, most of which were zinc finger genes (**Supplementary Figure 1**). Zinc finger proteins are the largest family of transcription factors in the human genome, and numerous studies have shown that mutations in zinc finger genes regulate the progression of cancer, including renal carcinoma (12). Mutations in Wilms tumor protein (WT1), a cys2-his2 zinc finger transcription factor, were found in both renal tumors and have been reported to cause a wide spectrum of renal and extrarenal manifestations concerning urogenital development and the development of tumors (13, 14). Moreover, mutations in the driver gene CREBBP were detected in both the right kidney and neck lesions, which is involved in the process of head and neck squamous cell carcinoma (HNSCC) as a chromatin regulator (15). In addition, we performed clonal evolution analysis to examine the heterogeneity of tumor evolution in different lesions. Bilateral nephropathy and neck lesions share some subclones, and there are a number of genetic mutations in these subclones, suggesting that these lesions may originate from a common ancestor and that there is a possibility of seeding evolution between them. Increasing research indicates that molecular technology can be used to aid correct diagnosis. Indeed, the morphological diversity occasionally observed in different parts of the same tumor, as well as the morphological diversity between the primary tumor and metastases, and even between different metastases of the same tumor, was consistent with the genetic differences observed between the primary and metastatic tumors (16). These events are in line with the recognized concept that cancer and its disseminated cells are affected by both normal and malignant cells in local and distant microenvironments (17, 18). The heterogeneity within the tumor is caused by the influence of genetic changes and the tumor microenvironment, both of which may reflect the variability of morphological characteristics (19).

We attempted to find an appropriate term to describe this phenomenon. The term “synchronous metastasis” refers to the simultaneous occurrence of metastasis and the primary tumor, while “metachronous” refers to the subsequent development of metastasis. We reported a case of bilateral RCC with different histological types between the primary, contralateral and neck metastatic tumors, but they shared the same clones respectively. This case suggests that genetic testing is critical for accurate diagnosis and treatment of patients with bilateral RCC and multiple lesions. Comparative molecular analysis will also help to better understand the potential role of genetic changes in the pathogenesis of RCC.

## Data Availability Statement

The raw data supporting the conclusions of this article will be made available by the authors, without undue reservation.

## Ethics Statement

Written informed consent was obtained from the individual(s) for the publication of any potentially identifiable images or data included in this article.

## Author Contributions

JD and FS: designed the study. JD, XH, and JZ: treated the patient and collected the data. CF, XY, WH, and CW: collected and analyzed the data. JD, CF, and XY: wrote the original draft. JZ and FS: reviewed and edited the manuscript. All authors contributed to the article and approved the submitted version.

## Funding

This research was funded by the National Natural Science Foundation of China (Grant number 81972494) and Scientific Research Project of Shanghai Health Committee (202040018).

## Conflict of Interest

The authors declare that the research was conducted in the absence of any commercial or financial relationships that could be construed as a potential conflict of interest.

## Publisher’s Note

All claims expressed in this article are solely those of the authors and do not necessarily represent those of their affiliated organizations, or those of the publisher, the editors and the reviewers. Any product that may be evaluated in this article, or claim that may be made by its manufacturer, is not guaranteed or endorsed by the publisher.

## Supplementary Material

Supplementary Figure 1 The genetic mutational landscape of 3 tumor tissues (The figure shows the top 50 genes with mutation frequency). Tkl, left kidney tumor; Tkr, right kidney tumor; Tn, neck tumor.
