# Supplementary figures and images for: Case Report: Rare Case of Synchronous Neck Metastasis From Metachronous Bilateral Renal Cell Carcinoma
Source: Front Oncol. 2021 Jul 27;11:677714. doi: 10.3389/fonc.2021.677714 (PMC8354027; doi:10.3389/fonc.2021.677714)

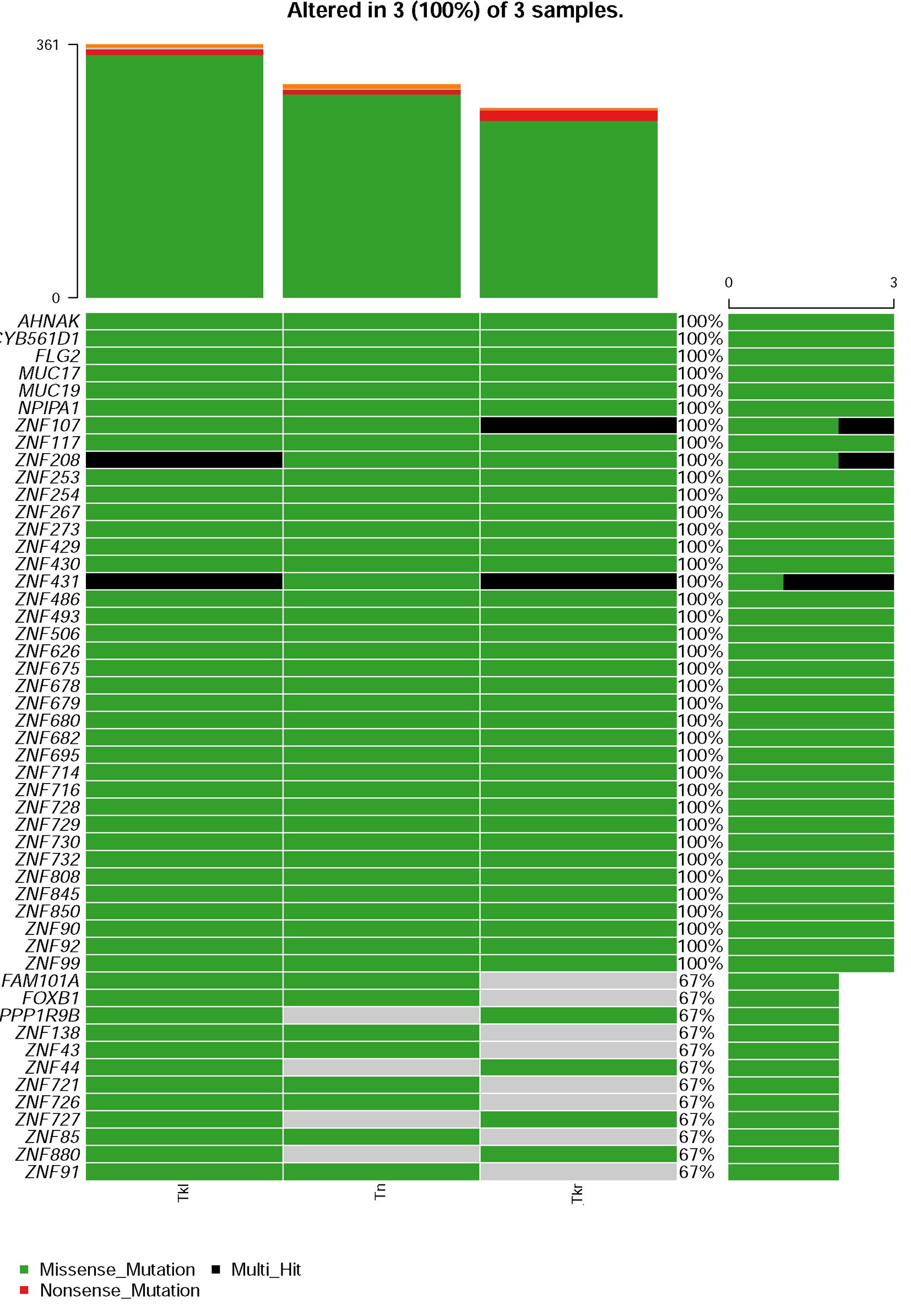

Supplement: Supplementary Figure 1 — The genetic mutational landscape of 3 tumor tissues (The figure shows the top 50 genes with mutation frequency). Tkl, left kidney tumor; Tkr, right kidney tumor; Tn, neck tumor. [file Image_1.tif]
